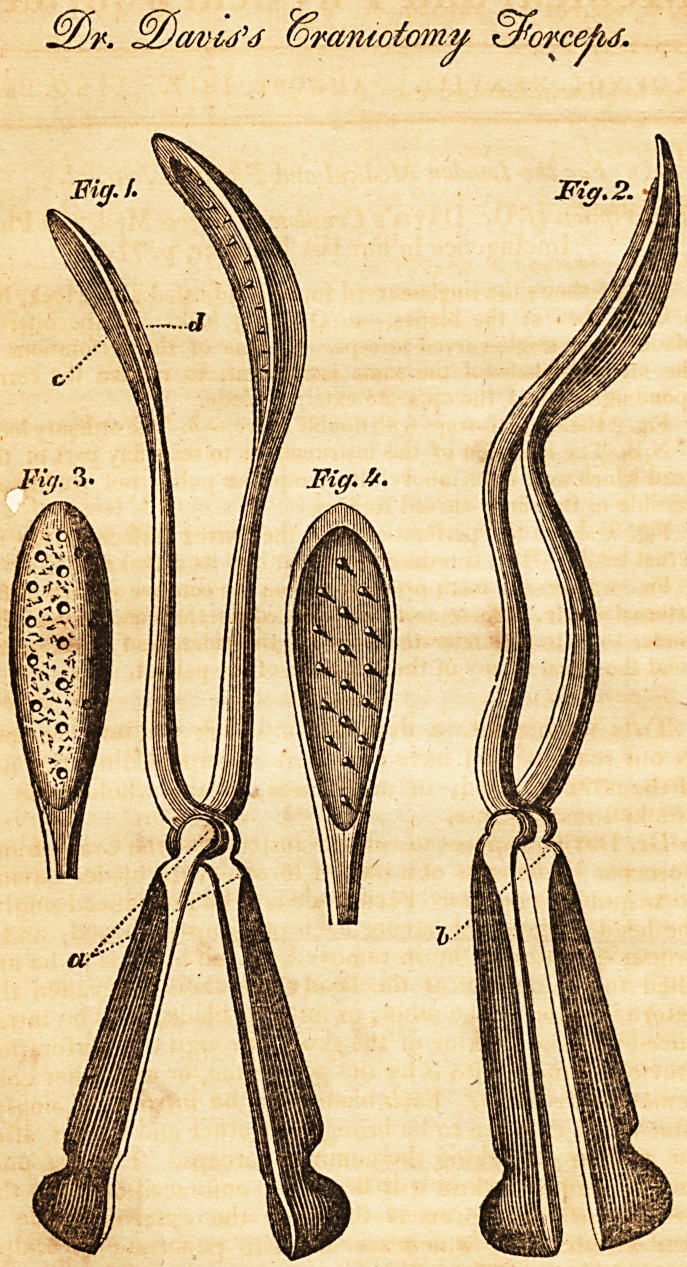# Description of Dr. Davis's Craniotomist

**Published:** 1817-08

**Authors:** 


### Dr. Davis's Craniotomy Forceps.

THE LONDON

### Medical and Physical Journal.

2 OF VOL. XXXVIII.] AUGUST, 1817. [NO. 222.

*For the London Medical and Physical Journal.*

### *Description of* Dr. DAVIS's *Craniotomist* (see Med. and Phil. Intelligence in our last Number, p. 77.)

FIG. 1 shews the single-curved forceps, adjusted at the lock, but a little open at the blades.—*a.* Ordinary lock. *c.* The internal blade of the single-curved forceps. *d.* One of the perforations in the internal blade of the same instrument, to receive the corresponding tooth of the opposite external blade.

Fig. 2 shews the forceps with double curve.—*b.* The ordinary lock.

N.B. The intention of this instrument is to seize any part of the head which may hitch above the symphysis pubis, not readily accessible to the single-curved forceps.

Fig. 3 shews the perforations into the convex surface of the internal blade. This is reduced to about half its actual size.

Fig. 4 shews the teeth projecting from the concave surface of the external blade. These teeth are buried in the concavity of the blade, so as to be under the level of the sides, and therefore beyond the reach of any of the soft parts of the patient.

---

THIS instrument, a drawing of which we now present to our readers, will have the effect of superseding the use of the crotchet, and, in most cases, of the whole tribe of crooked instruments.

Dr. Davis proposes to call the instrument the Craniotomy Forceps. It consists of a pair of forceps with blades having corresponding curves. Each blade is to be introduced singly, the head of the child having been previously opened, and a proper quantity of brain removed. One blade is to be applied to the outside of the head: this should be called the external blade. The other, or internal blade, is to be introduced into the interior of the skull, through the perforation previously made into it by the perforator, or any other convenient instrument. Each blade is to be introduced singly, after which they are to be brought together and locked, after the manner of locking the common forceps. Thus, a portion of scalp and skull will be firmly embraced between the two blades. To prevent slipping, the external blade is armed with teeth, which are made to penetrate the scalp; but which are so situated that they cannot possibly come in contact with any part of the mother. The opposite blade is

 bored bored with perforations corresponding with the teeth of the former.—See the engraving of the instruments, two forms of which are represented, adapted to different cases of distortion and different stages of the delivery.

Simplicity of structure is the great recommendation of the instrument in question, while with this simplicity it possesses great power, is extremely easy of application, and is without danger to the mother.

Dr. Davis was led to the improvement by the frequent calls made upon him to assist in cases to which the instrument is applicable, and by a conviction of the objections which oppose themselves to the plans hitherto pursued and practised in these instances of distressing emergency.

## Figures and Tables

**Fig. 1. Fig. 2. Fig. 3. Fig. 4. f1:**